# Multiparametric MRI radiomics combined with clinical-radiologic features improves preoperative prediction of muscle invasion in bladder cancer

**DOI:** 10.3389/fonc.2026.1949830

**Published:** 2026-10-26

**Authors:** Lei Qu, Zhihui Qu, Xuerong Ye, Gang Yuan, Liran Wang

**Affiliations:** 1 Department of Radiology, PLA Rocket Force Characteristic Medical Center, Beijing, China; 2 Department of Urology, PLA Rocket Force Characteristic Medical Center, Beijing, China; 3 Department of Radiology, Qianjiang Central Hospital, Qianjiang, Hubei, China

**Keywords:** bladder cancer, multiparametric MRI, muscle invasion, nomogram, radiomics, temporal validation, VI-RADS

## Abstract

**Background:**

Accurate preoperative differentiation of muscle-invasive bladder cancer (MIBC) from non-muscle-invasive bladder cancer (NMIBC) is central to treatment selection. We evaluated whether a nomogram integrating multiparametric MRI (mpMRI) radiomics, VI-RADS, and routinely recorded clinical-radiologic features improves prediction of muscle invasion.

**Methods:**

This single-center retrospective study included 350 consecutive patients with histologically confirmed bladder urothelial carcinoma who underwent preoperative 3.0-T mpMRI between January 2019 and December 2024. Patients were split chronologically into a training cohort (n = 245) and a temporal validation cohort (n = 105). Radiomics features were extracted from T2-weighted imaging, diffusion-weighted imaging, and apparent diffusion coefficient maps. Reproducibility filtering, univariable screening, collinearity pruning, and least-absolute-shrinkage-and-selection-operator regression were performed using the training cohort only. Clinical-radiologic, radiomics, and combined logistic models were evaluated by discrimination, calibration, Brier score, and decision curve analysis.

**Results:**

MIBC was present in 78 of 245 training patients (31.8%) and 33 of 105 validation patients (31.4%). Rad-score, VI-RADS score, tumor size, hydronephrosis, and absence of a tumor stalk remained independently associated with muscle invasion. The combined nomogram achieved AUCs of 0.929 (95% CI 0.895-0.963) in training and 0.897 (95% CI 0.839-0.955) in validation, compared with 0.708 for the clinical-radiologic model, 0.798 for VI-RADS, and 0.838 for the radiomics model in validation. Among the 59 patients with a research VI-RADS score of exactly 3, the combined nomogram achieved AUCs of 0.871 (95% CI 0.751-0.991) in training and 0.835 (95% CI 0.625-0.999) in validation; the pooled descriptive AUC was 0.864 (95% CI 0.764-0.964). Because VI-RADS was constant within this subgroup, an informative VI-RADS-alone ROC curve could not be estimated. Validation calibration intercept and slope for the combined model were 0.06 (95% CI -0.20 to 0.32) and 0.91 (95% CI 0.70-1.13), respectively.

**Conclusion:**

A combined mpMRI radiomics-clinical-radiologic nomogram showed strong temporal-validation performance for preoperative MIBC prediction, with an exploratory signal of utility in VI-RADS 3 lesions. External multicenter validation is required before clinical implementation.

## 1 Introduction

Bladder cancer is the ninth most commonly diagnosed malignancy worldwide, with approximately 614–000 new cases and 220–000 deaths estimated for 2022, and incidence rates that are roughly fourfold higher in men than in women (1, 2). Modeling based on demographic projections suggests that the global burden of bladder cancer cases and deaths could increase by approximately 73% and 87% by 2040, respectively, underlining the need for refined preoperative stratification tools that can be embedded in routine practice (1).

From a clinical management perspective, the most consequential decision at diagnosis is whether the tumor has invaded the detrusor muscle, because patients with NMIBC are typically managed with transurethral resection of bladder tumor (TURBT) followed by intravesical therapy and surveillance, whereas MIBC ordinarily warrants radical cystectomy or trimodal bladder-preservation therapy with neoadjuvant chemotherapy (3, 4). The 2024 European Association of Urology (EAU) guidelines on NMIBC and the AUA/ASCO/SUO MIBC guideline (amended 2024) both emphasize that misclassification at this junction may lead either to undertreatment of muscle-invasive disease or to unnecessary radical surgery, with substantial consequences for survival and quality of life (3–5).

Conventional preoperative work-up combines cystoscopy, cross-sectional imaging and TURBT. However, even with deep biopsy, TURBT-based staging is limited by sampling errors and is associated with non-trivial procedural risk and cost, and a substantial proportion of high-risk T1 patients are upstaged at radical cystectomy (6). To standardize the use of bladder mpMRI, Panebianco and colleagues introduced the Vesical Imaging-Reporting and Data System (VI-RADS) in 2018 (7). Subsequent meta-analyses have shown VI-RADS to be reproducible and to provide pooled sensitivities of approximately 88-90% and specificities of 80-85% for predicting muscle invasion at a cut-off of >= 3, with interobserver agreement that is generally substantial (8–10). Nevertheless, VI-RADS scores of 3 are considered a “gray zone”, and performance varies with reader experience and tumor location (11, 12).

Radiomics, which extracts high-dimensional quantitative descriptors of intensity, texture and shape from medical images, offers a complementary approach by capturing heterogeneity that may not be discernible to the human eye (13, 14). Previous studies have shown that mpMRI- or CT-based radiomics signatures can distinguish MIBC from NMIBC, and several investigations have already combined radiomics with VI-RADS, including analyses focused on VI-RADS 3 lesions (15–17). More recent work has explored deep learning, multi-task learning, and DCE-MRI-derived approaches with encouraging performance (18–20). Accordingly, the contribution of the present study is not the novelty of combining radiomics and VI-RADS per se, but the evaluation of this strategy in a comparatively large consecutive cohort with chronological temporal validation, blinded research VI-RADS reassessment, comprehensive discrimination and calibration assessment, and integration of routinely available clinical-radiologic variables.

We therefore retrospectively interrogated our institutional PACS and uro-oncology databases, which contain prospectively recorded but retrospectively extracted information on consecutive bladder cancer patients evaluated under an unchanged mpMRI protocol since 2019. Our objectives were threefold: (1) to derive an mpMRI-based radiomics signature using T2WI, DWI and ADC features extracted from already-acquired clinical scans; (2) to integrate the Rad-score with VI-RADS and routinely recorded clinical-radiologic variables into a logistic-regression nomogram; and (3) to evaluate, in a temporally separated validation cohort, discrimination, calibration, clinical utility, predictor redundancy, scanner robustness, and performance in the diagnostically uncertain VI-RADS 3 subgroup. Given the retrospective, single-center design, all results are framed as associations rather than evidence of causal superiority.

## 2 Materials and methods

### 2.1 Study design and ethical approval

#### 2.1.1 Study design

This was a single-center retrospective observational study conducted at a tertiary teaching hospital. All clinical, imaging and pathological data analyzed in this study were retrospectively retrieved from the institutional electronic medical record system, the PACS and the prospectively maintained departmental uro-oncology registry, which had been populated as part of routine clinical care between January 2019 and December 2024. No additional imaging examinations, biospecimens, questionnaires or clinical interventions were arranged for the purposes of this study; all data points reflected decisions taken under the standard institutional bladder cancer pathway by the multidisciplinary team in real time. Reporting followed the TRIPOD framework for prediction-model studies, with CLAIM and IBSI-aligned terminology and radiomics reporting where applicable.

#### 2.1.2 Ethical approval

The study protocol was reviewed and approved by the Institutional Ethics Review Board of the affiliated tertiary teaching hospital (Ethics Committee approval reference number LC2026077), which granted a waiver of written informed consent given the retrospective nature of data extraction, the absence of any contact with patients and the use of de-identified records. The formal ethics-committee letter granting the waiver of informed consent, together with the data-protection-impact assessment and the de-identification protocol, has been retained at the institutional research-management office and is available on reasonable request for verification by the editorial office. All patient identifiers were irreversibly anonymized before analysis, and the study was conducted in accordance with the principles of the Declaration of Helsinki and applicable local data-protection regulations.

### 2.2 Patient population

#### 2.2.1 Eligibility criteria

Eligibility criteria were applied retrospectively to the institutional registry. Inclusion criteria were as follows: (1) adult patients (≥ 18 years of age) with histopathologically confirmed urothelial carcinoma of the bladder; (2) availability of preoperative 3.0-T mpMRI performed at our institution within four weeks before TURBT or radical cystectomy as part of the routine clinical pathway; (3) availability of complete T2WI, DWI and ADC sequences with diagnostic image quality; and (4) availability of definitive histopathological staging. Exclusion criteria were as follows: (1) prior intravesical therapy, pelvic radiotherapy or radical cystectomy; (2) tumor with non-urothelial histology, mixed variant histology requiring different management, or pure carcinoma *in situ*; (3) MRI artifacts severe enough to preclude segmentation (e.g., motion or metallic artifacts from pelvic implants); (4) tumor diameter < 5 mm on T2WI, which falls below the recommended VI-RADS threshold for reliable scoring; and (5) incomplete clinical or pathological data in the electronic medical record.

#### 2.2.2 Cohort splitting

Of 412 patients initially identified through the registry, 62 were excluded (28 because of severe MRI artifacts or incomplete sequences, 19 because of non-urothelial or pure carcinoma-*in-situ* histology, 10 because of prior treatment and 5 because of incomplete clinical or pathological data), leaving a final analytical cohort of 350 patients. No additional patient was excluded for tumor diameter < 5 mm because none of the otherwise eligible cases met that criterion; the patient-flow accounting is summarized in **Supplementary Figure S1**. The cohort was split chronologically rather than by random sampling so as to better mimic prospective deployment: patients who underwent mpMRI between January 2019 and June 2023 (n = 245) served as the training cohort, and those imaged between July 2023 and December 2024 (n = 105) served as the temporal validation cohort. Across the entire study period, the institutional MRI hardware (Magnetom Skyra and Verio, Siemens Healthineers, both with stable software versions), the bladder mpMRI acquisition protocol, the surgical pathway algorithm and the WHO/ISUP histopathological grading and TNM staging criteria remained unchanged; major updates affected neither sequence parameters nor pT-classification rules. Two minor scanner-software service upgrades occurred (May 2021 and August 2023) but were vendor-validated as not altering quantitative DWI or ADC behavior. Group allocation therefore reflected calendar order of routine clinical care rather than randomization. Among the final 350 patients, all variables entering model development were complete, and no statistical imputation was performed.

### 2.3 MRI acquisition and image archiving

#### 2.3.1 Scanners and sequences

All examinations were retrospectively retrieved from PACS and had originally been performed on one of two 3.0-T MRI scanners (Magnetom Skyra or Verio, Siemens Healthineers, Erlangen, Germany) with a 32-channel phased-array body coil according to the same institutional bladder mpMRI protocol, which has remained essentially unchanged since 2019 and is consistent with VI-RADS technical recommendations (7). The protocol included (1) axial, sagittal and coronal high-resolution T2-weighted turbo-spin-echo sequences; (2) axial DWI with at least three b-values (typically 0, 800 and 1–400 s/mm^2^) and an automatically reconstructed ADC map; and (3) axial dynamic contrast-enhanced (DCE) T1-weighted gradient-echo imaging after intravenous administration of a standard-dose macrocyclic gadolinium-based contrast agent. Patient preparation, including moderate bladder filling, followed departmental standard operating procedures. Scanner-specific cohort distribution and outcome prevalence are reported in **Supplementary Table S5**. The acquisition framework documented for each scanner is summarized in **Supplementary Table S9**, including field strength, coil configuration, sequence classes, DWI b-value scheme, ADC reconstruction and DCE acquisition. Both scanners followed the same institutional protocol framework throughout the study period.

#### 2.3.2 Image quality control

Image quality was reassessed at the time of retrospective extraction by two abdominal radiologists working independently and blinded to clinical outcomes. Examinations were excluded only if they failed objective quality criteria (severe motion artifacts, geometric distortion of the bladder, or missing sequences). No study-specific intersequence registration or additional DWI distortion-correction algorithm was applied during the retrospective analysis; archived clinical reconstructions were used. The ADC maps were automatically reconstructed from their corresponding DWI acquisitions. Spatial correspondence of tumor VOIs across T2WI, DWI and ADC was maintained by simultaneous cross-reference to the other sequences and to anatomic landmarks during slice-by-slice delineation. No additional imaging was acquired or reprocessed for the study.

### 2.4 VI-RADS scoring and clinical variables

#### 2.4.1 VI-RADS scoring

VI-RADS scores generated as part of routine structured radiology reporting since 2019 (the “clinical-report VI-RADS”) were retrieved from PACS for descriptive comparison only. For the present analysis, two radiologists with eight and twelve years of pelvic MRI experience independently re-reviewed all cases on a dedicated workstation, blinded to histopathology and to each other, and assigned a research VI-RADS score (1–5) according to the original criteria proposed by Panebianco et al. (7). Disagreements between the two research readers were resolved by consensus. Agreement between the clinical-report VI-RADS and the consensus research VI-RADS was excellent (weighted Cohen κ = 0.86, 95% CI 0.81–0.91; complete numerical agreement in 81.4% of cases, all discordances within ± 1 category). Because re-evaluation was uniformly applied across the whole study population, the final analysis used the consensus research VI-RADS rather than the original clinical report to minimize heterogeneity introduced by case-by-case reading conditions, and this design choice is explicitly stated to support reproducibility. Inter-reader agreement between the two research radiologists was quantified using the weighted Cohen κ statistic. When multiple lesions were present, the lesion with the highest VI-RADS score was retained as the index lesion.

#### 2.4.2 Clinical-radiologic and demographic variables

Clinical-radiologic and demographic variables were extracted from the institutional electronic medical record and PACS by two trained data abstractors using a predefined extraction form. The variables retrieved included: (1) demographic data (age and sex); (2) smoking status (never, former, current); (3) presenting symptom (gross hematuria, microscopic hematuria, irritative voiding symptoms, incidental finding); (4) the presence of preoperative hydronephrosis on either imaging report or surgical record; (5) tumor size, defined as the largest diameter measured on T2WI by the radiology reading; (6) tumor multifocality (single versus multiple lesions); (7) tumor location (trigone, lateral wall, posterior wall, dome or anterior wall); (8) the presence of a tumor stalk on mpMRI; and (9) urine cytology category. All variables had been documented during routine pre-treatment work-up and were extracted in their original recorded form, without *post-hoc* redefinition.

### 2.5 Tumor segmentation and radiomics feature extraction

#### 2.5.1 Region-of-interest delineation

Whole-tumor volumes of interest (VOIs) were manually delineated on the axial T2WI, on the high-b-value DWI and on the ADC map using the open-source ITK-SNAP software (version 4.0; https://www.itksnap.org). For each patient, one abdominal radiologist (eight years of experience) performed an initial slice-by-slice segmentation, which was subsequently reviewed and, where necessary, refined by a second senior radiologist (twelve years of experience). Both readers were blinded to the histopathological outcome. The term VOI throughout the radiomics workflow refers to the tumor VOI rather than the entire bladder. When the bladder wall was indistinct on a single sequence, the radiologists cross-referenced the other sequences and anatomic landmarks to maintain spatially consistent tumor delineation across T2WI, DWI and ADC. To assess segmentation reproducibility, a reproducibility subsample of 50 cases was drawn exclusively from the training cohort (n = 245) by computer-generated stratified random sampling, with stratification on the muscle-invasion outcome to preserve the cohort prevalence (approximately one-third MIBC). No validation-cohort cases were used for ICC estimation, and the 50 cases remained part of the training cohort for all downstream modeling steps. These cases were re-segmented by the first reader after a four-week interval and independently by a third radiologist.

#### 2.5.2 Image preprocessing and feature extraction

Before feature extraction, all images were resampled to a standardized voxel spacing of 1 × 1 × 3 mm³ using B-spline interpolation. PyRadiomics intensity normalization was performed before discretization using I_norm = 100 × (I - μ)/σ, where μ and σ were calculated from the voxels of the input image volume supplied to PyRadiomics; normalization was therefore image-based rather than computed from the tumor mask. The normalizeScale parameter was 100, so the normalized intensities did not have unit variance. A fixed bin width of 25 was then applied for gray-level discretization. The resulting median numbers of occupied gray-level bins were 24 (IQR 21-28; range 14-39) for T2WI, 22 (IQR 19-27; range 13-38) for DWI, and 23 (IQR 20-27; range 14-41) for ADC (**Supplementary Table S8**), confirming that texture matrices were not generated from only one or two intensity bins. These image-level preprocessing operations were distinct from the subsequent patient-level standardization of extracted radiomics features used for model development and were implemented with PyRadiomics version 3.1.0 (21); the complete parameter file is provided as Supplementary File 1. DCE-MRI was used as an integral component of research VI-RADS assessment but was not included in the radiomics pipeline because DCE radiomics can be sensitive to contrast-injection timing, temporal resolution and enhancement-phase selection, whereas T2WI, high-b-value DWI and ADC were consistently available across the cohort and supported a more standardized feature-extraction workflow. Exclusion of DCE from radiomics may omit complementary perfusion-related information and is addressed as a limitation. For each of the three radiomics sequences, four feature classes were calculated: (1) first-order statistics; (2) shape-based three-dimensional features; (3) gray-level co-occurrence matrix (GLCM), gray-level run-length matrix (GLRLM), gray-level size-zone matrix (GLSZM), gray-level dependence matrix (GLDM) and neighboring gray-tone difference matrix (NGTDM) texture features; and (4) the same texture features computed after wavelet decomposition and Laplacian-of-Gaussian (σ = 1.0, 2.0, 3.0) filtering. A total of 1–423 features were extracted from each MRI sequence, yielding 4–269 candidate radiomics features per patient.

### 2.6 Feature selection and Rad-score construction

#### 2.6.1 Reproducibility filtering

To retain only reproducible features, the intra-class correlation coefficient (ICC) was computed across the 50 paired re-segmentations (all drawn from the training cohort) for both intra- and inter-reader pairs. Features with both ICCs ≥ 0.80 were retained; the remainder were discarded. This step removed approximately one third of the candidate features, yielding a robust pool for subsequent statistical selection.

#### 2.6.2 LASSO selection

Within the training cohort only, surviving features underwent univariate screening using the Mann–Whitney U test (P < 0.05). Highly collinear features (Spearman |ρ> 0.85) were further pruned, retaining the feature with the stronger univariate association. Before LASSO modeling, each remaining feature was standardized across patients using the mean and standard deviation estimated exclusively from the training cohort; the same training-derived transformation was applied unchanged to the temporal validation cohort. The standardized features were entered into a least-absolute-shrinkage-and-selection-operator (LASSO) logistic-regression model with ten-fold cross-validation, in which the optimal regularization parameter λ was selected using the one-standard-error rule (log λ = −2.30, corresponding to a binomial deviance of 0.80; the LASSO coefficient path and cross-validation deviance curve are presented in **Figure 1**). Eleven features with non-zero coefficients were retained: four from T2WI, four from ADC and three from DWI. The identity and LASSO coefficient of each retained feature are reported in **Table 1**. A continuous Rad-score was computed for each patient as the linear combination of the selected standardized features weighted by their LASSO coefficients, plus the model intercept. No validation-cohort data were used for feature filtering, standardization, regularization-parameter selection or coefficient estimation.

**Figure 1:**
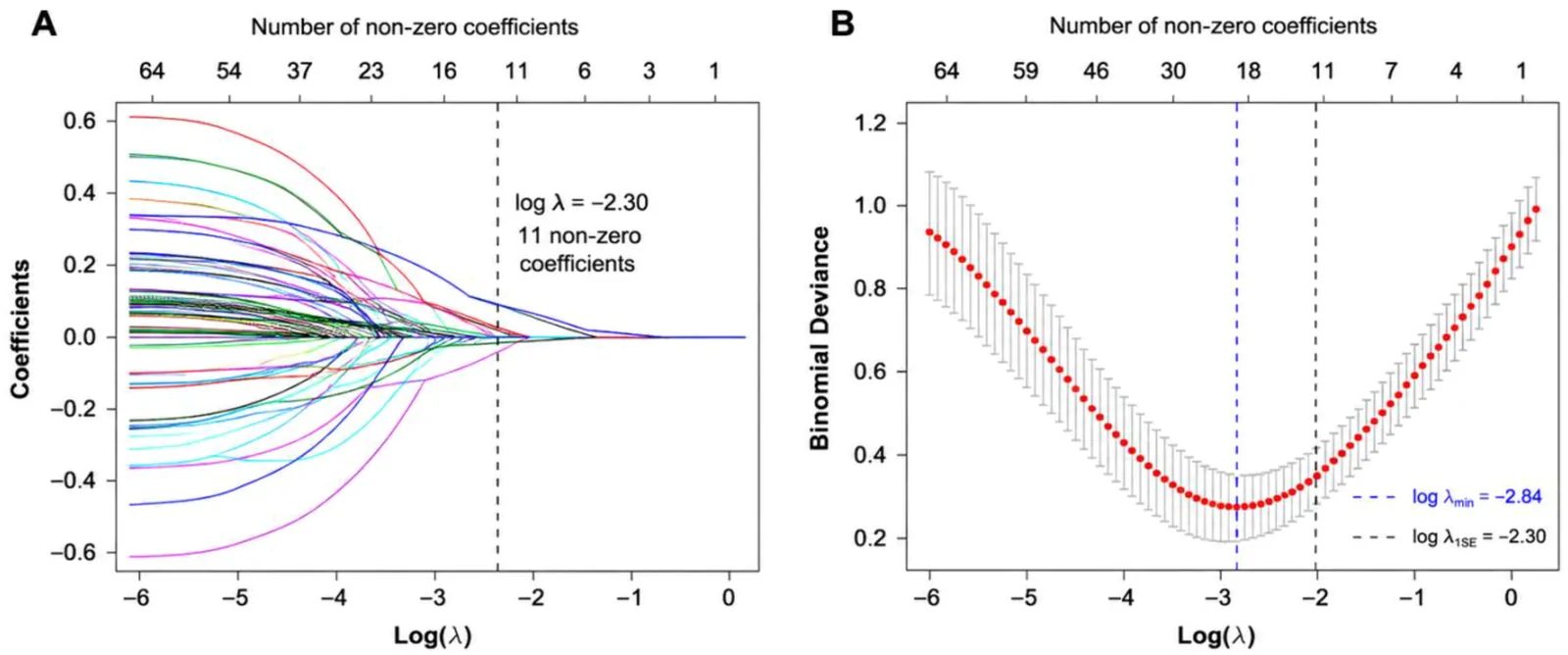
LASSO feature selection in the training cohort (n = 245). **(A)** Coefficient path showing the trajectory of each candidate feature as a function of log(λ); the vertical dashed line marks the value selected by the one-standard-error rule (log λ = −2.30), at which eleven features retain non-zero coefficients. **(B)** Ten-fold cross-validation binomial deviance with error bars; the blue dashed line marks log λmin (−2.84) and the black dashed line log λ1SE (−2.30). The numbers at the top of each panel indicate the number of features with non-zero coefficients at the corresponding log(λ).

**Table 1:** The 11 radiomics features retained by LASSO regression and used to compute the Rad-score.

ID	Sequence	Feature class	Feature name	LASSO coefficient
F1	T2WI	GLCM	wavelet-LHL_glcm_ClusterShade	0.418
F2	T2WI	First-order	log-sigma-2.0_firstorder_Skewness	−0.327
F3	T2WI	GLSZM	original_glszm_SizeZoneNonUniformity	0.241
F4	T2WI	Shape (3D)	original_shape_SurfaceVolumeRatio	0.183
F5	ADC	First-order	original_firstorder_10Percentile	−0.452
F6	ADC	GLCM	wavelet-HLL_glcm_Correlation	0.276
F7	ADC	NGTDM	log-sigma-3.0_ngtdm_Contrast	0.214
F8	ADC	GLRLM	original_glrlm_RunVariance	−0.157
F9	DWI (b = 1 400)	First-order	original_firstorder_Energy	0.362
F10	DWI (b = 1 400)	GLDM	wavelet-LLL_gldm_DependenceVariance	0.198
F11	DWI (b = 1 400)	GLCM	log-sigma-1.0_glcm_Imc1	−0.139
Intercept	—	—	—	0.087

GLCM, gray-level co-occurrence matrix; GLDM, gray-level dependence matrix; GLRLM, gray-level run-length matrix; GLSZM, gray-level size-zone matrix; NGTDM, neighboring gray-tone difference matrix; T2WI, T2-weighted imaging; ADC, apparent diffusion coefficient; DWI, diffusion-weighted imaging. Rad-score = 0.087 + Σ(βi × normalized feature value), with the βi values listed above.

#### 2.6.3 Collinearity, redundancy, and volume-sensitivity analysis

To assess residual collinearity and the potential confounding effect of tumor volume, we first computed a complete 11 × 11 Spearman correlation matrix among the LASSO-selected features. For each feature, the Spearman correlation coefficient and the coefficient of determination (R^2^) from a separate univariable ordinary-least-squares regression were calculated against both the maximum tumor diameter measured on T2WI and the corresponding sequence-specific three-dimensional ROI volume derived from the segmentation mask. The reported R^2^ values therefore quantify linear explained variance and were not obtained by squaring the Spearman coefficient. For the volume-adjusted sensitivity analysis, each selected feature was regressed on log-transformed ROI volume using the training cohort only. The intercept and slope estimated in the training cohort were applied unchanged to the validation cohort, and the resulting residuals were standardized using the training-cohort mean and standard deviation. A volume-adjusted Rad-score was then reconstructed using the original LASSO coefficients. To evaluate conceptual overlap among predictors in the combined model, variance inflation factors (VIFs) and Spearman correlations were calculated for the Rad-score, VI-RADS, tumor size, hydronephrosis, and tumor-stalk status. Three reduced sensitivity models were additionally fitted in the training cohort and applied unchanged to the validation cohort: Rad-score + VI-RADS + hydronephrosis; Rad-score + tumor size + tumor-stalk status + hydronephrosis; and Rad-score + VI-RADS. The volume-adjusted combined model and the reduced models were evaluated without validation-cohort updating. Finally, in a univariable ordinary-least-squares regression in which both a radiomics feature and tumor size are standardized across the same patients, the regression slope equals the Pearson correlation coefficient; similar standardized slopes alone therefore do not establish that multiple radiomics features were deterministically generated from tumor size.

### 2.7 Pathological assessment

All histopathological reports were retrospectively retrieved from the institutional pathology information system. The reference standard for muscle invasion was detrusor muscle infiltration (>= pT2) on the highest-quality definitive pathological assessment available for each patient, with radical-cystectomy pathology taking precedence over TURBT when both were available. Among the 350 included patients, 252 (72.0%) had a radical-cystectomy specimen: 107 had both TURBT and subsequent cystectomy, and 145 had cystectomy as the definitive index pathology in the study database, meaning that no preceding TURBT report contributed to the study reference standard. The remaining 98 patients (28.0%) had TURBT as the sole pathological reference. Among the 107 patients with paired TURBT and cystectomy assessments, muscle-invasion classification was concordant in 88 (82.2%) and discordant in 19 (17.8%): 17 were classified as NMIBC on TURBT and upstaged to MIBC at cystectomy, whereas 2 showed the reverse pattern. The median interval between MRI and the definitive pathological procedure was 12 days (IQR 8–19 days), with 12 days (IQR 8-18) in training and 13 days (IQR 8-20) in validation (P = 0.514). Fourteen TURBT specimens had inadequate detrusor-muscle representation. No patient was classified as MIBC solely on the basis of an inadequately sampled TURBT specimen; subsequent cystectomy or documented follow-up evidence of muscle invasion was required for an MIBC classification. An additional sensitivity analysis excluded all 14 cases with inadequate detrusor sampling. A separate sensitivity analysis restricted performance evaluation to the 252 cystectomy-confirmed patients. Because selection for radical cystectomy was related to routine multidisciplinary assessment and disease severity, the cystectomy-restricted analysis was interpreted as a robustness check rather than a complete solution to verification bias. Repeat TURBT status was not captured as a separate structured analytical variable and therefore its frequency and independent contribution to stage clarification were not quantified. When a radical-cystectomy specimen was available, cystectomy remained the definitive reference standard irrespective of the preceding TURBT history. Cystectomy indication was not among the predefined variables extracted for the analytical dataset, so indication-specific frequencies are not quantified; these limitations are addressed in the Discussion. No re-review of pathology slides was performed.

### 2.8 Model construction and statistical analysis

#### 2.8.1 Model construction

Within the training cohort, three primary logistic-regression models were built. The first was a clinical-radiologic model, in which candidate demographic and clinical-radiologic variables with P < 0.10 on univariable analysis were entered into a multivariable logistic regression with backward stepwise selection based on the Akaike Information Criterion (AIC). The second was a radiomics-only model, in which the Rad-score was the sole predictor. The third was a combined model that incorporated the Rad-score, the VI-RADS score and the independent clinical-radiologic predictors retained in the first model. The combined model was visualized as a nomogram constructed using the rms package in R. The volume-adjusted, reduced-predictor, scanner-adjusted, cystectomy-restricted, and inadequate-sampling sensitivity analyses were treated as secondary robustness analyses rather than additional primary models.

#### 2.8.2 Statistical analysis

Continuous variables were summarized as mean ± standard deviation or median (interquartile range), as appropriate, and compared between groups using the independent-samples t-test or the Mann-Whitney U test. Categorical variables were summarized as counts and percentages and compared using the χ^2^ test or Fisher’s exact test. To control for potential confounding introduced by the chronological split, training and validation cohorts were compared on each baseline variable, and any variable showing a between-cohort difference at P < 0.10 was forced into the multivariable logistic-regression models as a covariate in a sensitivity analysis. Discrimination was assessed using the AUC of ROC curves with 95% confidence intervals derived by 1 000-replicate bootstrapping; AUCs were compared using the DeLong test where both predictors varied within the evaluated sample. Calibration was assessed using calibration curves, the Hosmer-Lemeshow test, Brier score, and logistic recalibration intercept and slope with 95% confidence intervals. Clinical utility was evaluated using DCA over threshold probabilities of 0.10-0.80 to characterize model behavior across a broad range of risk preferences; this range was not interpreted as a direct treatment threshold for radical cystectomy. The probability threshold of 0.50 was used only as a conventional descriptive threshold. Validation-cohort sensitivity, specificity, positive predictive value and negative predictive value were additionally reported at illustrative thresholds of 0.30, 0.40 and 0.50 (**Supplementary Table S7**). A predefined subgroup analysis was performed in patients with a research VI-RADS score of exactly 3. Because VI-RADS is constant by definition within this subgroup, an informative VI-RADS-alone ROC curve cannot be estimated; subgroup ROC analyses therefore compared the radiomics and combined model results descriptively, with training and temporal-validation estimates reported separately. Scanner type was added as an additional covariate in a sensitivity model. Two-sided P-values < 0.05 were considered statistically significant. All analyses were performed in R (version 4.3.2; R Foundation for Statistical Computing, Vienna, Austria) and Python (version 3.11).

## 3 Results

### 3.1 Baseline characteristics

A total of 350 patients (mean age 66.4 ± 11.2 years; 281 men, 69 women) were included. Muscle invasion was histologically confirmed in 78 of 245 (31.8%) patients in the training cohort and 33 of 105 (31.4%) patients in the validation cohort. Baseline demographic and tumor-related variables were closely balanced between cohorts (**Table 2**). Scanner distribution was also nearly identical: Skyra examinations accounted for 150/245 (61.2%) training cases and 64/105 (61.0%) validation cases, with Verio accounting for the remainder (P = 0.967). MIBC prevalence was similar within scanner strata (Skyra: 48/150 [32.0%] in training and 20/64 [31.3%] in validation; Verio: 30/95 [31.6%] and 13/41 [31.7%], respectively; **Supplementary Table S5**). Among the 107 patients with both TURBT and cystectomy pathology, 88 (82.2%) were concordant and 19 (17.8%) were discordant for muscle-invasion status, including 17 TURBT-to-cystectomy upstagings from NMIBC to MIBC and 2 reverse classifications (**Supplementary Table S6**). Patients with MIBC were older and were more likely to present with hydronephrosis, larger tumor diameter, sessile morphology and higher VI-RADS scores (**Table 2** and **Supplementary Table S1**).

**Table 2:** Baseline clinical, imaging and pathological characteristics of the training and validation cohorts.

Variable	Training (n = 245)	Validation (n = 105)	P value
Age, years, mean ± SD	66.1 ± 11.4	67.0 ± 10.8	0.482
Male sex, n (%)	197 (80.4)	84 (80.0)	0.928
Smoking status, n (%)			0.612
Never	92 (37.6)	42 (40.0)	
Former	78 (31.8)	30 (28.6)	
Current	75 (30.6)	33 (31.4)	
Gross hematuria, n (%)	189 (77.1)	82 (78.1)	0.842
Hydronephrosis, n (%)	61 (24.9)	27 (25.7)	0.870
Tumor size, cm, median (IQR)	2.6 (1.7–3.8)	2.7 (1.8–4.0)	0.405
Multifocality, n (%)	84 (34.3)	38 (36.2)	0.728
Tumor stalk present, n (%)	144 (58.8)	60 (57.1)	0.768
VI-RADS score, n (%)			0.591
1	47 (19.2)	18 (17.1)	
2	82 (33.5)	33 (31.4)	
3	39 (15.9)	20 (19.0)	
4	44 (18.0)	21 (20.0)	
5	33 (13.5)	13 (12.4)	
Reference pathology, n (%)			0.713
Radical cystectomy	175 (71.4)	77 (73.3)	
TURBT only	70 (28.6)	28 (26.7)	
Muscle invasion (≥ pT2), n (%)	78 (31.8)	33 (31.4)	0.940
Scanner type, n (%)			0.967
Magnetom Skyra	150 (61.2)	64 (61.0)	
Magnetom Verio	95 (38.8)	41 (39.0)	

SD, standard deviation; IQR, interquartile range; VI-RADS, Vesical Imaging-Reporting and Data System; TURBT, transurethral resection of bladder tumor.

### 3.2 Radiomics signature and Rad-score distribution

The intra- and inter-reader ICCs of the candidate features were excellent (median ICCs 0.91 and 0.88, respectively), and 2–743 of 4–269 features met the >= 0.80 reproducibility threshold. Following normalization with normalizeScale = 100 and fixed-bin-width discretization, the median occupied gray-level bin counts were 24 for T2WI, 22 for DWI and 23 for ADC, with sequence-specific ranges of 14-39, 13–38 and 14-41, respectively (**Supplementary Table S8**). After univariate filtering and removal of highly collinear features, 64 features were entered into the LASSO selection step, of which 11 features (four T2WI, four ADC, three DWI) were retained at the optimal λ (log λ = -2.30, corresponding to a binomial deviance of 0.80 at ten-fold cross-validation; **Figure 1**). The full list of the 11 retained features, including the parent sequence, feature class, definition and LASSO coefficient, is reported in **Table 1**. The resulting Rad-score was significantly higher in the MIBC group than in the NMIBC group in both cohorts (P < 0.001 in each; **Figure 2**).

**Figure 2:**
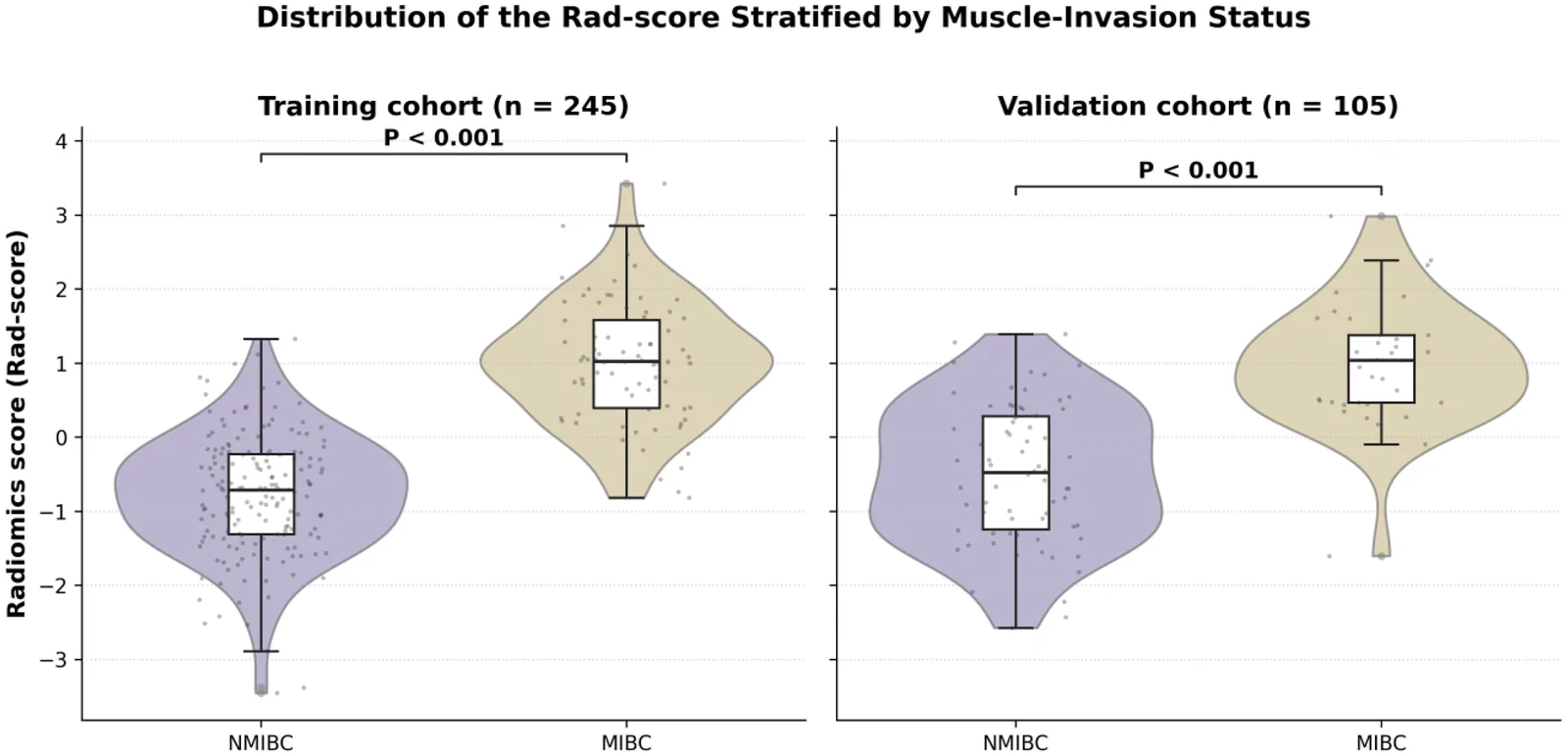
Distribution of the Rad-score stratified by muscle-invasion status in the training (left) and validation (right) cohorts. Violin plots show the underlying density, embedded box plots indicate the median and interquartile range, and individual points are overlaid as jittered dots. P-values were derived from the Mann–Whitney U test.

### 3.3 Univariable and multivariable analyses

Univariable logistic regression in the training cohort revealed that age, hydronephrosis, tumor size, the absence of a tumor stalk, the VI-RADS score and the Rad-score were all significantly associated with muscle invasion (**Table 3**). On multivariable analysis adjusting for collinearity, the Rad-score, VI-RADS score, tumor size, hydronephrosis and absence of a tumor stalk remained independent predictors. The complete logistic-regression equation of the combined nomogram model, including the intercept, coefficients (β), standard errors and 95% confidence intervals, is provided in **Table 4**. Inter-reader agreement for VI-RADS was substantial (weighted κ = 0.81), consistent with previously published estimates (10).

**Table 3:** Univariable and multivariable logistic regression analyses for muscle invasion in the training cohort (n = 245).

Variable	Univariable OR (95% CI)	P	Multivariable OR (95% CI)	P
Age (per 10 years)	1.31 (1.05–1.64)	0.018	1.18 (0.92–1.51)	0.187
Male sex	1.42 (0.79–2.55)	0.246	—	—
Current smoking	1.26 (0.78–2.04)	0.347	—	—
Hydronephrosis (yes)	4.92 (2.74–8.83)	< 0.001	2.84 (1.42–5.69)	0.003
Tumor size (per 1 cm)	1.62 (1.36–1.93)	< 0.001	1.31 (1.06–1.62)	0.013
Multifocality (yes)	1.46 (0.86–2.48)	0.158	—	—
Tumor stalk absent	3.27 (1.91–5.60)	< 0.001	2.16 (1.15–4.07)	0.017
VI-RADS score (per unit)	3.11 (2.36–4.10)	< 0.001	1.95 (1.40–2.71)	< 0.001
Rad-score (per unit)	4.78 (3.32–6.88)	< 0.001	3.42 (2.27–5.16)	< 0.001

OR, odds ratio; CI, confidence interval; VI-RADS, Vesical Imaging-Reporting and Data System.

**Table 4:** Complete coefficients of the combined-nomogram multivariable logistic regression model (training cohort, n = 245).

Predictor	β (SE)	OR (95% CI)	Wald χ^2^	P
Intercept	−6.85 (0.94)	—	53.10	< 0.001
Rad-score (per unit)	1.230 (0.207)	3.42 (2.27–5.16)	35.36	< 0.001
VI-RADS score (per unit)	0.668 (0.169)	1.95 (1.40–2.71)	15.62	< 0.001
Tumor size (per 1 cm)	0.270 (0.108)	1.31 (1.06–1.62)	6.27	0.013
Hydronephrosis (yes vs no)	1.044 (0.354)	2.84 (1.42–5.69)	8.70	0.003
Tumor stalk (absent vs present)	0.770 (0.323)	2.16 (1.15–4.07)	5.69	0.017

β, regression coefficient on the log-odds scale; SE, standard error of β; OR, odds ratio = exp(β); CI, confidence interval; VI-RADS, Vesical Imaging-Reporting and Data System. Model linear predictor: logit(P) = −6.85 + 1.230 × Rad-score + 0.668 × VI-RADS + 0.270 × tumor size (cm) + 1.044 × hydronephrosis (yes = 1) + 0.770 × tumor stalk absent (yes = 1). Likelihood-ratio χ^2^ = 178.4 (5 df), P < 0.001; Nagelkerke R^2^ = 0.624; Hosmer–Lemeshow χ^2^ = 6.21, P = 0.624.

### 3.4 Model performance

ROC analysis in the training cohort yielded AUCs of 0.848 (95% CI 0.798-0.898) for the clinical-radiologic model, 0.846 (95% CI 0.796-0.896) for the VI-RADS score, 0.911 (95% CI 0.874-0.948) for the radiomics model and 0.929 (95% CI 0.895-0.963) for the combined nomogram (**Figure 3**). In the validation cohort, the corresponding AUCs were 0.708 (95% CI 0.612-0.804), 0.798 (95% CI 0.716-0.880), 0.838 (95% CI 0.762-0.914) and 0.897 (95% CI 0.839-0.955), respectively. DeLong testing showed that the combined nomogram had higher AUC than the clinical-radiologic, VI-RADS and radiomics-only models in the training cohort (all P < 0.05) and than the clinical-radiologic and VI-RADS-only models in the validation cohort (P = 0.012 and P = 0.038, respectively); the difference between the combined and radiomics-only models in validation did not reach statistical significance (P = 0.118). At illustrative validation probability thresholds of 0.30, 0.40 and 0.50, the combined model yielded sensitivities of 90.9%, 87.9% and 84.8% and specificities of 73.6%, 80.6% and 86.1%, respectively; the corresponding PPV/NPV values are reported in **Supplementary Table S7**. In the 252-patient cystectomy-confirmed sensitivity subset, discrimination remained similar: the combined nomogram achieved AUCs of 0.924 (95% CI 0.884-0.964) in training and 0.892 (95% CI 0.815-0.969) in validation, while validation AUCs were 0.701 for the clinical-radiologic model, 0.790 for VI-RADS and 0.832 for radiomics (**Supplementary Table S2**). Detailed diagnostic metrics are summarized in **Table 5** and **Supplementary Table S2**.

**Figure 3:**
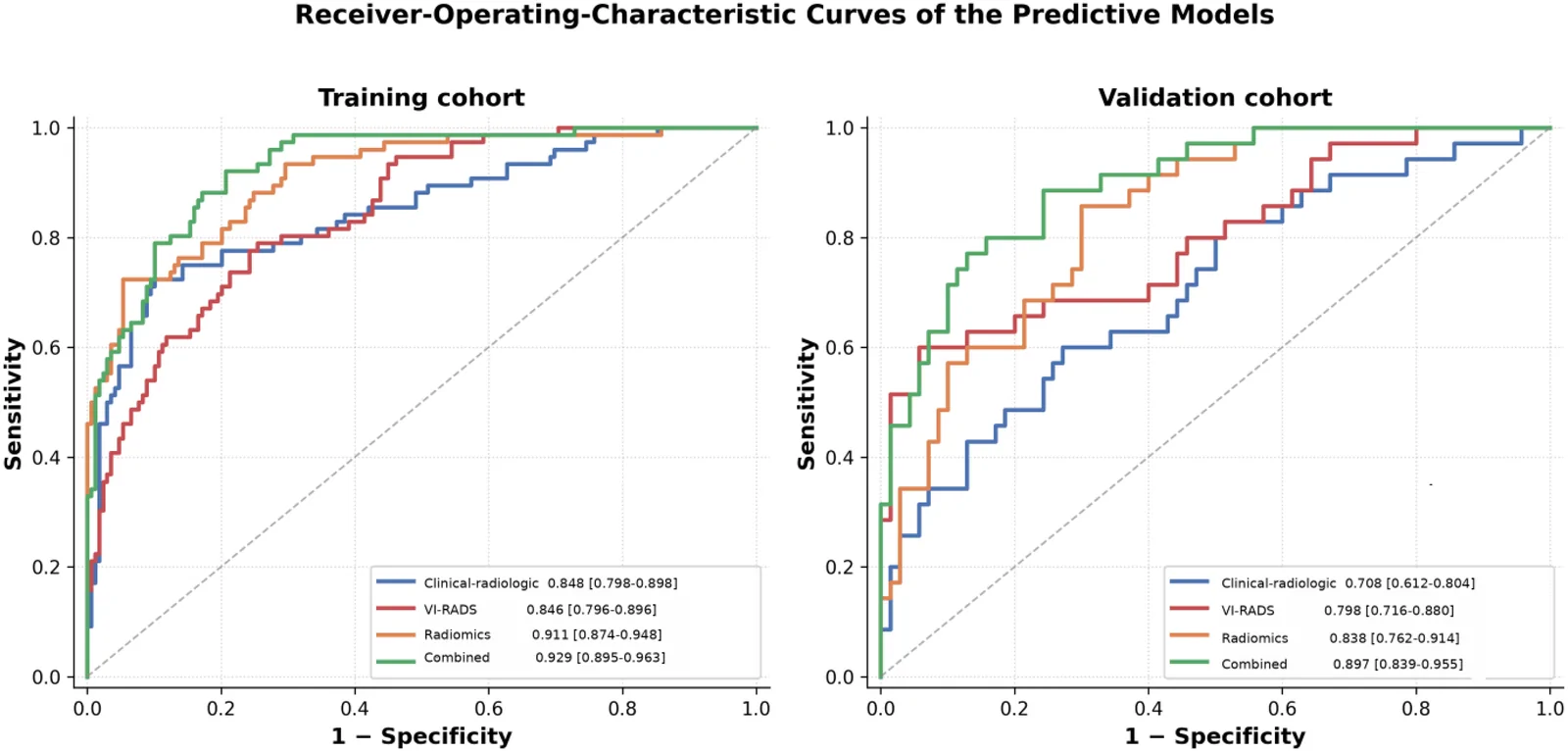
Receiver-operating-characteristic curves of the clinical-radiologic model, VI-RADS score, radiomics model and combined nomogram in the training (left) and validation (right) cohorts. Legends report AUC [95% CI] for each model.

**Table 5:** Diagnostic performance of the four models in the training and validation cohorts.

Model/cohort	AUC (95% CI)	Sensitivity	Specificity	Accuracy
Training cohort				
Clinical-radiologic	0.848 (0.798–0.898)	0.756	0.731	0.739
VI-RADS score	0.846 (0.796–0.896)	0.808	0.808	0.808
Radiomics	0.911 (0.874–0.948)	0.821	0.838	0.833
Combined nomogram	0.929 (0.895–0.963)	0.872	0.868	0.869
Validation cohort				
Clinical-radiologic	0.708 (0.612–0.804)	0.727	0.722	0.724
VI-RADS score	0.798 (0.716–0.880)	0.788	0.819	0.810
Radiomics	0.838 (0.762–0.914)	0.788	0.847	0.829
Combined nomogram	0.897 (0.839–0.955)	0.848	0.861	0.857

AUC, area under the receiver-operating-characteristic curve; CI, confidence interval; VI-RADS, Vesical Imaging-Reporting and Data System.

### 3.5 Subgroup analysis in the VI-RADS 3 “gray zone”

Fifty-nine patients had a research VI-RADS score of exactly 3: 39 in the training cohort (15 MIBC, 24 NMIBC) and 20 in the temporal validation cohort (7 MIBC, 13 NMIBC), yielding 22 MIBC events overall (37.3%). Because the research VI-RADS score was constant at 3 by definition within this subgroup, an informative ROC curve for VI-RADS alone could not be estimated. The combined nomogram achieved an AUC of 0.871 (95% CI 0.751-0.991) in the training subgroup and 0.835 (95% CI 0.625-0.999) in the validation subgroup; the corresponding radiomics-model AUCs were 0.816 (95% CI 0.684-0.948) and 0.779 (95% CI 0.562-0.996), respectively. For descriptive purposes, the pooled 59-patient subgroup yielded AUCs of 0.864 (95% CI 0.764-0.964) for the combined model and 0.812 (95% CI 0.702-0.922) for radiomics. At a conventional probability threshold of 0.50, combined-model sensitivity, specificity and accuracy were 80.0%, 83.3% and 82.1% in training and 85.7%, 76.9% and 80.0% in validation, respectively; pooled values were 81.8%, 81.1% and 81.4%. Because the validation subgroup contained only 20 patients and 7 MIBC events, these estimates are exploratory and hypothesis-generating.

### 3.6 Calibration of the combined nomogram

Calibration assessment complemented discrimination analysis. In the training cohort, calibration intercepts/slopes were 0.00/0.99 for the clinical-radiologic model, 0.01/1.01 for VI-RADS, -0.01/0.98 for radiomics and 0.00/0.97 for the combined nomogram. In temporal validation, the clinical-radiologic model showed a calibration intercept of -0.18 (95% CI -0.46 to 0.10) and a slope of 0.70 (95% CI 0.48-0.92), consistent with appreciable calibration deterioration. In contrast, the combined nomogram showed an intercept of 0.06 (95% CI -0.20 to 0.32) and a slope of 0.91 (95% CI 0.70-1.13); VI-RADS and radiomics validation slopes were 0.87 and 0.89, respectively (**Supplementary Table S3**). The combined-model calibration curves remained close to the ideal 45° line (**Figure 4**), with Hosmer-Lemeshow χ^2^ = 6.21 (P = 0.624) in training and χ^2^ = 7.04 (P = 0.532) in validation. Combined-model Brier scores were 0.092 and 0.105, respectively. The relatively wide validation confidence intervals are consistent with the limited number of MIBC events (n = 33).

**Figure 4:**
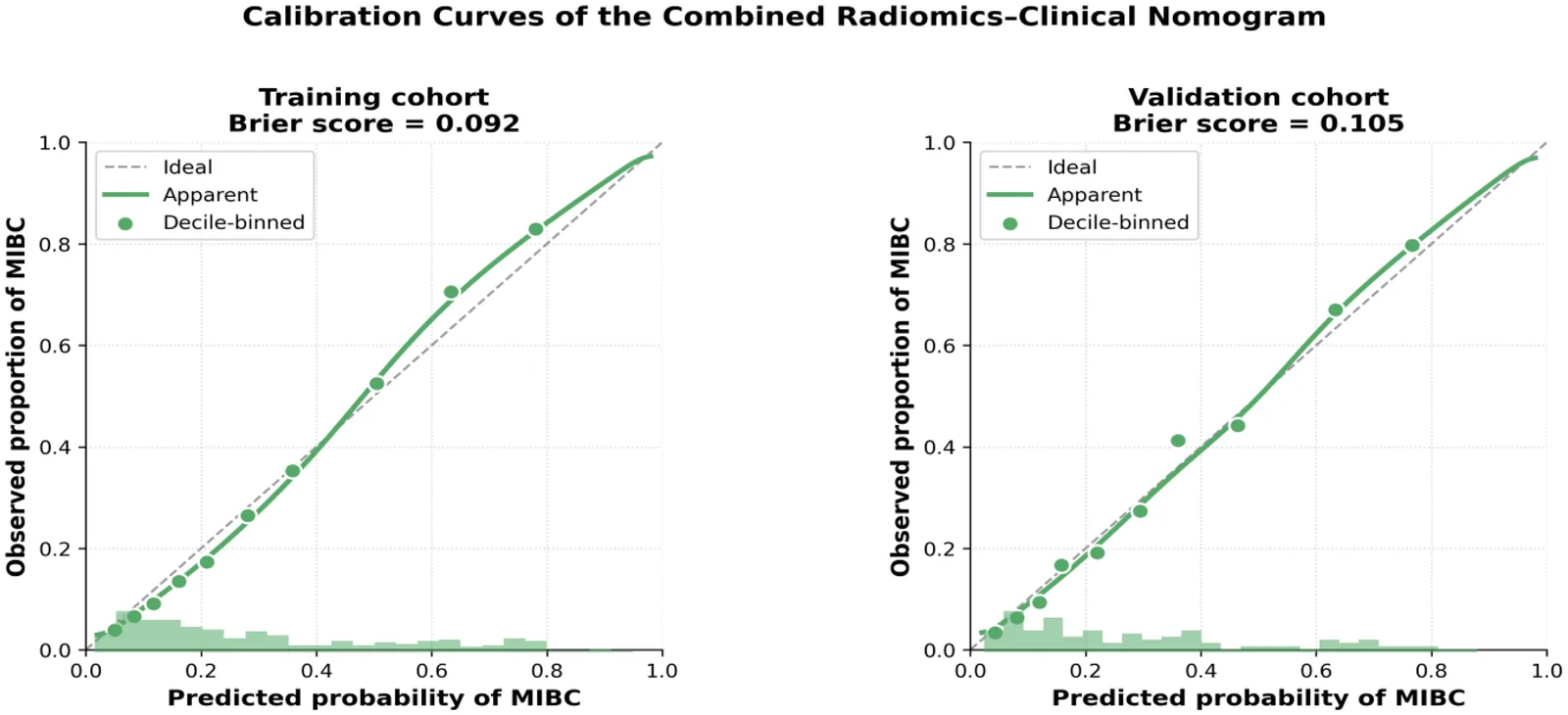
Calibration curves of the combined nomogram in the training and validation cohorts. Solid green lines depict the smoothed apparent calibration; dots indicate decile-binned observed proportions; dashed gray lines indicate ideal calibration. Histograms below each panel show the distribution of predicted probabilities.

### 3.7 Nomogram visualization

The combined model was rendered as a clinically oriented nomogram (**Figure 5**) incorporating the Rad-score, VI-RADS score, tumor size, hydronephrosis and tumor stalk. Consistent with the multivariable regression coefficients reported in **Table 4**, the Rad-score has the largest range of attainable Points (0 to 100), reflecting its largest β × range contribution, whereas the VI-RADS score, tumor size, hydronephrosis and tumor-stalk absence span maxima of approximately 36, 27, 14 and 10 Points, respectively. Each predictor’s value is mapped onto the upper ‘Points’ axis; the sum across predictors gives a Total points score that maps to the predicted probability of muscle invasion.

**Figure 5:**
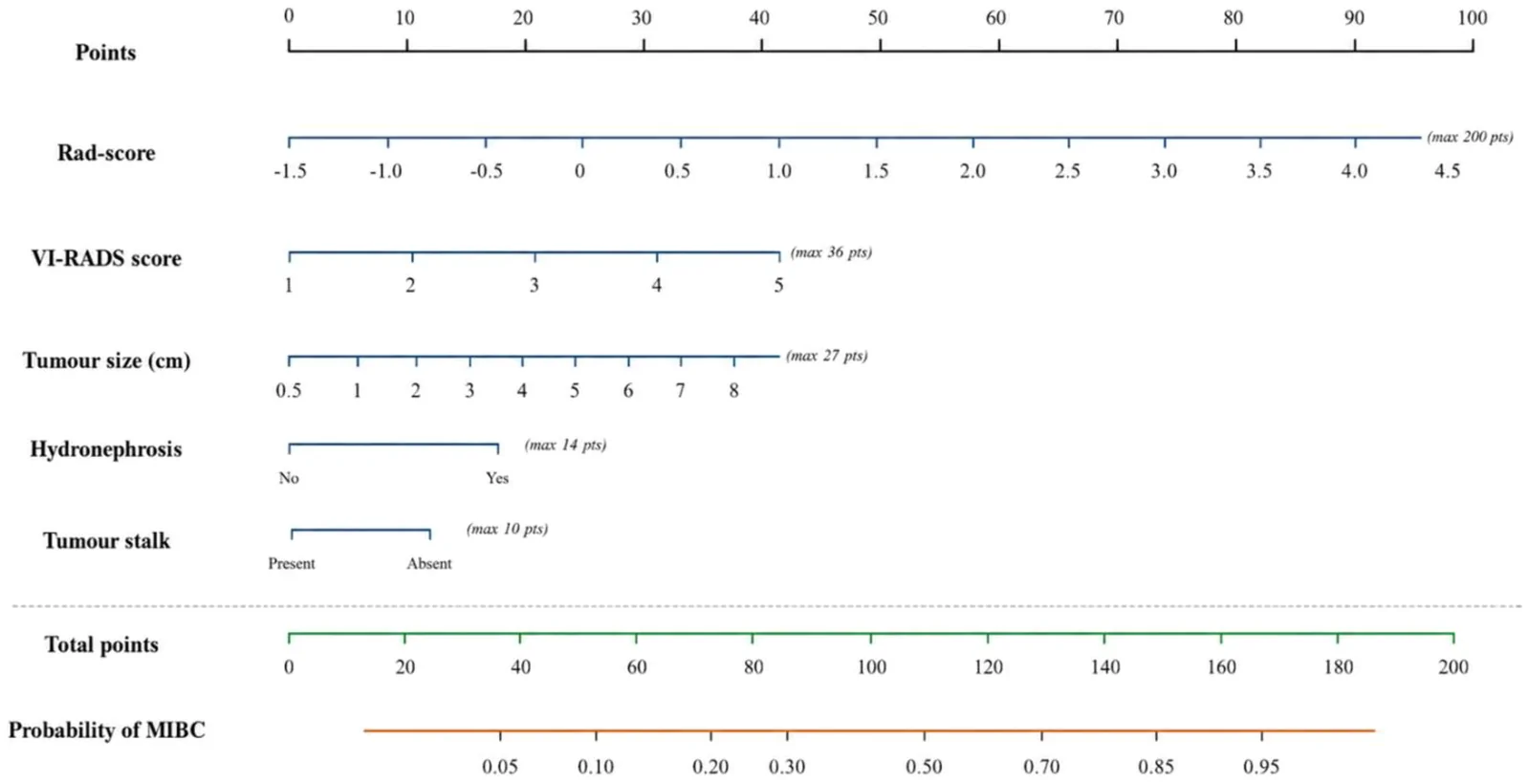
Nomogram for predicting muscle invasion in bladder cancer combining the Rad-score, VI-RADS score, tumor size, hydronephrosis and the presence of a tumor stalk. The Points scale of each predictor is proportional to its multivariable logistic-regression coefficient (**Table 4**), so that variables with stronger associations contribute a wider Points range. The corresponding regression equation is shown at the bottom of the figure.

### 3.8 Decision curve analysis

DCA showed that the combined nomogram was associated with greater net benefit than the treat-all, treat-none, clinical-radiologic, VI-RADS-only and radiomics-only strategies across much of the evaluated threshold-probability range in both cohorts (**Figure 6**). The 0.10-0.80 range was used to characterize model behavior over a broad spectrum of risk preferences and should not be interpreted as a direct indication for radical cystectomy. In clinical practice, a predicted probability from the nomogram would represent one component of multidisciplinary assessment together with cystoscopic, pathological and other clinical findings. The DCA is therefore interpreted as evidence of potential decision-support value rather than evidence that the model should independently determine treatment.

**Figure 6:**
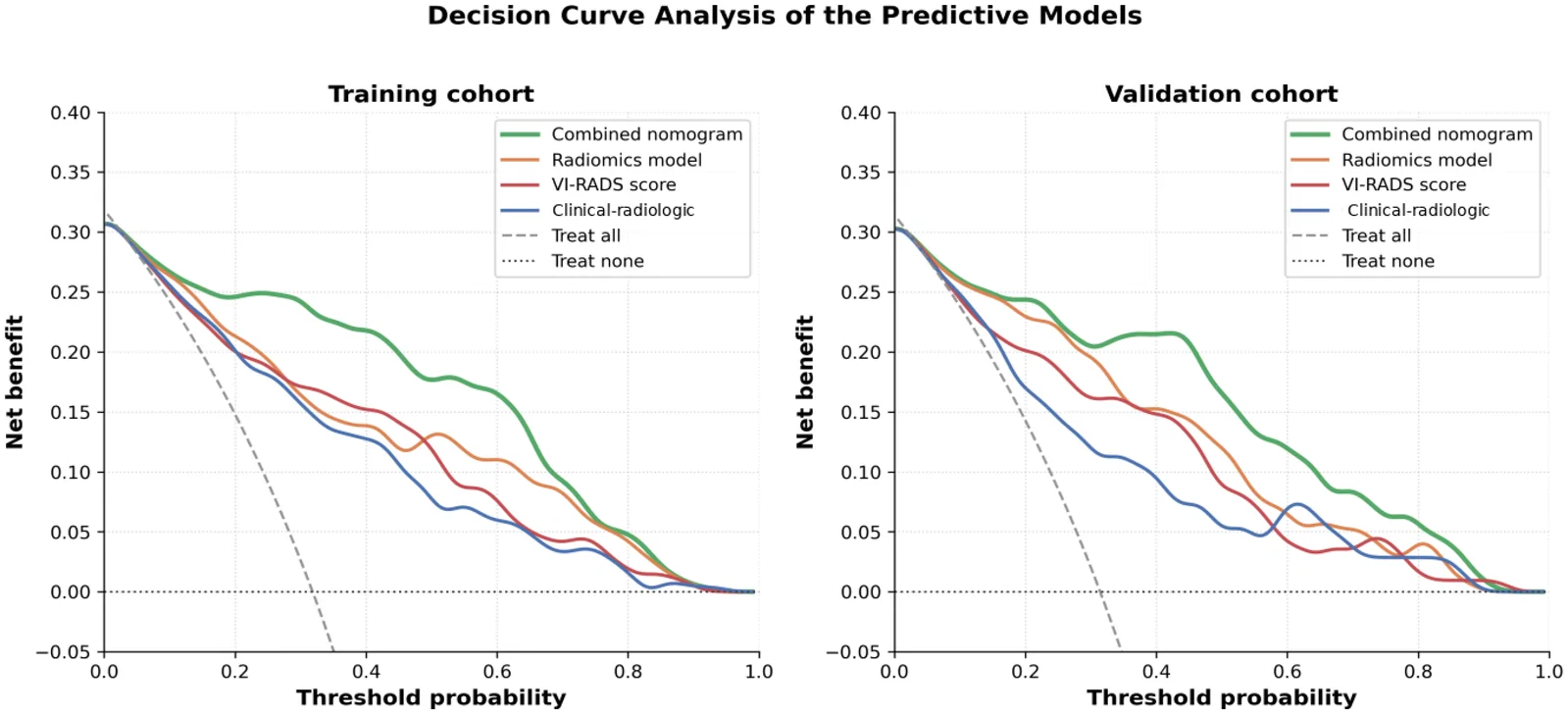
Decision curve analysis of the four models in the training and validation cohorts. The gray dashed line and dotted horizontal line correspond to the treat-all and treat-none strategies, respectively. The displayed threshold-probability range characterizes decision-support behavior and is not a stand-alone treatment threshold.

### 3.9 Collinearity, redundancy, and sensitivity analyses

Among the 11 selected radiomics features, the 11 × 11 Spearman correlation matrix showed a maximum absolute pairwise correlation of 0.62 and a median absolute correlation of 0.24; no feature pair exceeded the prespecified collinearity threshold of 0.85. Correlations with sequence-specific ROI volume ranged from -0.41 for original_shape_SurfaceVolumeRatio to 0.58 for original_firstorder_Energy, while R^2^ values from separate univariable linear regressions ranged from 0.04 to 0.31. The volume-adjusted combined nomogram retained AUCs of 0.903 (95% CI 0.866-0.940) in training and 0.881 (95% CI 0.818-0.944) in validation, and the volume-adjusted Rad-score remained independently associated with muscle invasion after adjustment for tumor size (OR 3.18, 95% CI 2.09-4.84, P < 0.001). For the combined-model predictors, VIFs were 1.74 for Rad-score, 1.89 for VI-RADS, 1.46 for tumor size, 1.22 for hydronephrosis and 1.37 for tumor-stalk absence. VI-RADS showed moderate Spearman correlations with the Rad-score (ρ = 0.48), tumor size (ρ = 0.43), tumor-stalk absence (ρ = 0.39) and hydronephrosis (ρ = 0.31). Reduced sensitivity models yielded validation AUCs of 0.886 for Rad-score + VI-RADS + hydronephrosis, 0.882 for Rad-score + tumor size + tumor-stalk status + hydronephrosis, and 0.884 for Rad-score + VI-RADS, compared with 0.897 for the full combined model (**Supplementary Table S4**). Thus, the predictors showed expected partial information overlap but no severe multicollinearity, and model discrimination did not depend on simultaneous inclusion of all overlapping variables. Scanner adjustment also had negligible influence: adding scanner type to the combined logistic model yielded AUCs of 0.930 in training and 0.896 in validation, while scanner itself was not independently associated with muscle invasion (β = 0.11; OR 1.12, 95% CI 0.70-1.79; P = 0.641; **Supplementary Table S5**). Finally, excluding all 14 patients with inadequate detrusor-muscle representation on TURBT left 336 patients and produced AUCs of 0.928 and 0.895 for the combined model in training and validation, respectively; corresponding clinical-radiologic, VI-RADS and radiomics AUCs were likewise essentially unchanged (**Supplementary Table S6**).

## 4 Discussion

In this single-center retrospective study of 350 patients with bladder urothelial carcinoma, a nomogram integrating an mpMRI radiomics signature with VI-RADS, tumor size, hydronephrosis and tumor-stalk status was associated with high discrimination and favorable calibration for preoperative muscle-invasion prediction. The combined nomogram achieved AUCs of 0.929 in training and 0.897 in temporal validation. Its validation calibration slope was 0.91, and its performance was materially preserved in cystectomy-confirmed, scanner-adjusted, volume-adjusted and inadequate-TURBT-sampling sensitivity analyses. These results describe predictive associations in a retrospective registry and do not establish clinical superiority or a treatment effect.

The present findings extend, rather than originate, the existing literature on combining radiomics with VI-RADS. Zheng and colleagues reported improved performance from combining an mpMRI radiomics signature with VI-RADS in a cohort of 185 patients (15), Zhang et al. described a CT-based radiomics nomogram (16), and Kabanov and colleagues reported a compact T2WI/ADC radiomics descriptor with multicohort validation (17). The main contribution of the current study is therefore its comparatively large consecutive cohort, chronological temporal validation, blinded consensus VI-RADS reassessment, explicit calibration and decision-curve evaluation, and sensitivity analyses addressing reference-standard uncertainty, scanner effects, volume dependence and predictor redundancy. The absolute performance estimates should nevertheless be interpreted in the context of single-center cohort selection and require external validation.

Several mechanistic considerations support the observed associations. Quantitative ADC values are inversely related to cell density, and ADC- and DWI-derived radiomics descriptors have previously been associated with biological aggressiveness, neoadjuvant chemotherapy response and muscle invasiveness in bladder cancer (22). Radiomics features derived from ADC and T2WI may therefore capture intratumoral heterogeneity that complements visual interpretation. Clinical-radiologic predictors such as preoperative hydronephrosis and sessile morphology are also clinically plausible markers of more advanced local disease (6, 23, 24). Their inclusion should be interpreted as integration of partially overlapping risk information rather than as evidence that each predictor represents an independent biological pathway.

The VI-RADS 3 analysis is best interpreted as exploratory. Within this subgroup all patients had the same research VI-RADS score by definition, so VI-RADS alone cannot generate an informative ROC curve. The combined model achieved AUCs of 0.871 in the 39-patient training subgroup and 0.835 in the 20-patient temporal-validation subgroup, whereas the radiomics model achieved 0.816 and 0.779, respectively. The pooled descriptive combined-model AUC was 0.864. These results are directionally consistent with the possibility that quantitative imaging and clinical-radiologic information can refine risk estimation in indeterminate VI-RADS 3 lesions (11, 12), but the validation subgroup contained only seven MIBC events and the confidence intervals were correspondingly wide. Meta-analytic evidence likewise shows substantial heterogeneity in VI-RADS performance across studies (8, 9, 25). Prospective multicenter evaluation is required before this subgroup observation can support clinical decision-making.

Clinically, preoperative estimation of muscle invasion may support multidisciplinary triage, but model probabilities should not be treated as stand-alone indications for radical treatment. Current bladder-cancer guidelines emphasize integration of imaging with cystoscopic, pathological and patient-level information (3, 4). Accordingly, the DCA threshold range of 0.10-0.80 in this study was used to characterize net-benefit behavior over a broad range of risk preferences rather than to define a surgical threshold. The illustrative 0.30, 0.40 and 0.50 operating points similarly describe diagnostic trade-offs and are not prespecified treatment cut-offs. Real-world VI-RADS-based pathways have also been evaluated as decision-support tools rather than autonomous treatment rules (26). This distinction is particularly important because treatment allocation in the present retrospective cohort was determined by routine multidisciplinary decisions rather than by the nomogram.

Beyond classical radiomics, deep-learning approaches based on T2WI or DCE-MRI have reported encouraging external performance (18, 19, 27). Handcrafted radiomics retains greater feature-level transparency but is sensitive to preprocessing, discretization and scanner-related choices. In the present workflow, PyRadiomics normalization used a scale factor of 100 before fixed-bin-width discretization, yielding median occupied bin counts of 22–24 rather than the extremely sparse binning that would follow unit-variance z-score normalization with a bin width of 25. The full preprocessing configuration is supplied to improve reproducibility. DCE was retained for VI-RADS assessment but excluded from the radiomics signature to avoid additional variability from contrast timing and phase selection; this choice may have omitted complementary perfusion information and should be tested in future multicenter work. Methodological frameworks such as METRICS emphasize transparent preprocessing, strict separation of training and validation data, and reproducibility assessment (13, 28).

Potential information overlap among radiomics, VI-RADS and morphology was examined explicitly. VI-RADS correlated moderately with the Rad-score, tumor size and tumor-stalk status, but all combined-model VIFs were below 2.0. Removing tumor size and tumor-stalk status reduced validation AUC from 0.897 to 0.886, whereas removing VI-RADS reduced it to 0.882; a Rad-score + VI-RADS model yielded 0.884. These analyses do not eliminate conceptual overlap, but they argue against severe multicollinearity or complete redundancy as the sole explanation for combined-model performance. Similarly, selected radiomics features retained partial volume dependence, particularly original_firstorder_Energy and original_shape_SurfaceVolumeRatio (21), yet volume-adjusted discrimination remained 0.881 in validation and the adjusted Rad-score remained independently associated with muscle invasion. These findings support robustness while preserving predictor overlap and residual volume dependence as methodological limitations.

Several important limitations should be considered. First, all data were collected retrospectively at a single tertiary center, and temporal validation within the same institution is not equivalent to external validation. Second, the clinical-radiologic model showed marked performance deterioration from training AUC 0.848 to validation AUC 0.708 and a validation calibration slope of 0.70; temporal variation and the use of univariable screening followed by backward stepwise selection may have contributed to coefficient instability. Third, the pathological reference standard combined TURBT and cystectomy. Paired assessments were discordant in 17.8% of patients, predominantly because TURBT understated subsequent cystectomy stage, and cystectomy-restricted analysis remains vulnerable to verification bias because the probability of undergoing cystectomy is related to disease severity. The high cystectomy proportion therefore reflects a selected tertiary surgical population rather than the expected treatment distribution of unselected NMIBC. Cystectomy indication and repeat TURBT status were not among the predefined structured variables extracted for the analytical dataset, so indication-specific frequencies and the independent role of repeat TURBT could not be quantified reliably. Fourth, 14 TURBT specimens had inadequate detrusor sampling; exclusion of these cases had negligible influence on model AUCs but cannot remove all outcome-misclassification risk. Fifth, two MRI scanners were used, although scanner distributions and MIBC prevalence were closely balanced, both followed the same documented acquisition framework, and scanner-adjusted discrimination was unchanged. Sixth, no study-specific intersequence registration or additional DWI distortion correction was applied, and manual cross-sequence VOI correspondence may retain residual spatial error. Seventh, DCE radiomics was not evaluated. Eighth, five patients with incomplete key data were excluded before analysis, and complete-case selection bias cannot be excluded. Ninth, manual segmentation remains time-consuming, although automated approaches may reduce this burden (29). Finally, the model may require recalibration in populations with different MIBC prevalence, referral patterns or acquisition protocols, and prospective multicenter evaluation is required before clinical implementation.

## 5 Conclusion

In this single-center retrospective study of 350 patients, a nomogram combining an mpMRI radiomics signature, VI-RADS and routinely recorded clinical-radiologic variables was associated with high discrimination, acceptable calibration and potential decision-support value for preoperative prediction of muscle invasion in bladder cancer. Performance remained similar across several sensitivity analyses, while the VI-RADS 3 subgroup showed an exploratory signal that requires larger independent validation. The retrospective, non-randomized, single-center design and the use of a temporal rather than external validation cohort limit generalizability; prospective multicenter validation and potential recalibration are required before clinical implementation.

## Data Availability

The original contributions presented in the study are included in the article/**Supplementary Material**. Further inquiries can be directed to the corresponding authors.

## Supplementary material

Supplementary Table 1 Baseline comparison between non–muscle-invasive bladder cancer (NMIBC) and muscle-invasive bladder cancer (MIBC) groups in the pooled cohort (n = 350).
