# Supplementary material for: Multiparametric MRI radiomics combined with clinical-radiologic features improves preoperative prediction of muscle invasion in bladder cancer
Source: Front Oncol. 2026 Oct 26;16:1949830. doi: 10.3389/fonc.2026.1949830 (PMC13745341; doi:10.3389/fonc.2026.1949830)
Supplement: Supplementary file 1 [file SupplementaryFile1.docx]

**Supplementary Material**

*Table S1. Baseline comparison between non–muscle-invasive bladder cancer (NMIBC) and muscle-invasive bladder cancer (MIBC) groups in the pooled cohort (n = 350).*

| **Variable** | **NMIBC (n = 239)** | **MIBC (n = 111)** | **P value** |
| --- | --- | --- | --- |
| Age, years, mean ± SD | 64.3 ± 11.0 | 70.6 ± 10.8 | < 0.001 |
| Male sex, n (%) | 187 (78.2) | 94 (84.7) | 0.158 |
| Current smoking, n (%) | 67 (28.0) | 41 (36.9) | 0.090 |
| Hydronephrosis, n (%) | 37 (15.5) | 51 (45.9) | < 0.001 |
| Tumour size, cm, median (IQR) | 2.1 (1.5–3.0) | 3.9 (2.8–5.1) | < 0.001 |
| Multifocality, n (%) | 78 (32.6) | 44 (39.6) | 0.197 |
| Tumour stalk present, n (%) | 166 (69.5) | 38 (34.2) | < 0.001 |
| VI-RADS ≥ 3, n (%) | 63 (26.4) | 107 (96.4) | < 0.001 |
| Rad-score, median (IQR) | −0.71 (−1.34 to −0.05) | 1.04 (0.42 to 1.62) | < 0.001 |
| Reference pathology, n (%) |  |  | < 0.001 |
| Radical cystectomy | 143 (59.8) | 109 (98.2) |  |
| TURBT only | 96 (40.2) | 2 (1.8) |  |

*SD, standard deviation; IQR, interquartile range; VI-RADS, Vesical Imaging-Reporting and Data System; TURBT, transurethral resection of bladder tumour.*

Table S2. Diagnostic performance in the radical-cystectomy-confirmed sensitivity subset.

| **Model** | **AUC (95% CI)** | **Sensitivity** | **Specificity** | **Accuracy** |
| --- | --- | --- | --- | --- |
| Training cohort, n = 175 |  |  |  |  |
| Clinical-radiologic | 0.842 (0.785-0.899) | 76.3% | 73.7% | 74.9% |
| VI-RADS | 0.840 (0.783-0.897) | 80.3% | 80.8% | 80.6% |
| Radiomics | 0.905 (0.860-0.950) | 81.6% | 83.8% | 82.9% |
| Combined nomogram | 0.924 (0.884-0.964) | 86.8% | 85.9% | 86.3% |
| Validation cohort, n = 77 |  |  |  |  |
| Clinical-radiologic | 0.701 (0.584-0.818) | 72.7% | 70.5% | 71.4% |
| VI-RADS | 0.790 (0.688-0.892) | 78.8% | 81.8% | 80.5% |
| Radiomics | 0.832 (0.742-0.922) | 78.8% | 84.1% | 81.8% |
| Combined nomogram | 0.892 (0.815-0.969) | 84.8% | 86.4% | 85.7% |

Performance metrics were recalculated within the cystectomy-confirmed subset using the same model definitions as the primary analysis; the analysis was used as a robustness check for reference-standard uncertainty.

Table S3. Calibration metrics of the four models in the training and temporal validation cohorts.

| **Model** | **Calibration intercept (95% CI)** | **Calibration slope (95% CI)** | **Brier score** |
| --- | --- | --- | --- |
| Training cohort |  |  |  |
| Clinical-radiologic | 0.00 (-0.19 to 0.19) | 0.99 (0.82-1.17) | 0.141 |
| VI-RADS | 0.01 (-0.18 to 0.20) | 1.01 (0.84-1.18) | 0.137 |
| Radiomics | -0.01 (-0.19 to 0.17) | 0.98 (0.83-1.13) | 0.108 |
| Combined nomogram | 0.00 (-0.18 to 0.18) | 0.97 (0.83-1.12) | 0.092 |
| Validation cohort |  |  |  |
| Clinical-radiologic | -0.18 (-0.46 to 0.10) | 0.70 (0.48-0.92) | 0.185 |
| VI-RADS | -0.04 (-0.31 to 0.23) | 0.87 (0.64-1.11) | 0.155 |
| Radiomics | 0.03 (-0.24 to 0.30) | 0.89 (0.67-1.12) | 0.129 |
| Combined nomogram | 0.06 (-0.20 to 0.32) | 0.91 (0.70-1.13) | 0.105 |

Table S4. Predictor redundancy and reduced-model sensitivity analyses.

| **Analysis** | **Result** | **Training AUC** | **Validation AUC** |
| --- | --- | --- | --- |
| VIF: Rad-score | 1.74 |  |  |
| VIF: VI-RADS | 1.89 |  |  |
| VIF: tumor size | 1.46 |  |  |
| VIF: hydronephrosis | 1.22 |  |  |
| VIF: tumor-stalk absence | 1.37 |  |  |
| Spearman: VI-RADS vs Rad-score | ρ = 0.48 |  |  |
| Spearman: VI-RADS vs tumor size | ρ = 0.43 |  |  |
| Spearman: VI-RADS vs stalk absence | ρ = 0.39 |  |  |
| Spearman: VI-RADS vs hydronephrosis | ρ = 0.31 |  |  |
| Full combined model | Reference | 0.929 | 0.897 |
| Rad-score + VI-RADS + hydronephrosis | Reduced model | 0.918 | 0.886 |
| Rad-score + tumor size + stalk + hydronephrosis | Reduced model | 0.920 | 0.882 |
| Rad-score + VI-RADS | Reduced model | 0.915 | 0.884 |

VIF, variance inflation factor. Reduced models were fitted in the training cohort and evaluated in the temporal validation cohort without validation-cohort updating.

Table S5. Scanner distribution, outcome prevalence, and scanner-adjusted sensitivity analysis.

| **Scanner / model** | **Training n** | **Training MIBC n (%)** | **Validation n** | **Validation MIBC n (%)** |
| --- | --- | --- | --- | --- |
| Magnetom Skyra | 150 | 48 (32.0%) | 64 | 20 (31.3%) |
| Magnetom Verio | 95 | 30 (31.6%) | 41 | 13 (31.7%) |
| Total | 245 | 78 (31.8%) | 105 | 33 (31.4%) |

Scanner distribution was nearly identical between training and validation cohorts (Skyra 61.2% vs 61.0%, P = 0.967). When scanner type was added to the combined model, scanner was not independently associated with muscle invasion (β = 0.11; OR 1.12, 95% CI 0.70-1.79; P = 0.641). The scanner-adjusted combined model achieved AUCs of 0.930 in training and 0.896 in validation, compared with 0.929 and 0.897 for the primary combined model.

Table S6. Pathological-reference concordance and sensitivity analysis excluding inadequate detrusor sampling.

| **Component** | **Training / paired result** | **Validation / sensitivity result** | **Overall / note** |
| --- | --- | --- | --- |
| Paired TURBT and cystectomy: NMIBC -> NMIBC |  |  | 38 |
| Paired TURBT and cystectomy: MIBC -> MIBC |  |  | 50 |
| Paired TURBT and cystectomy: NMIBC -> MIBC |  |  | 17 |
| Paired TURBT and cystectomy: MIBC -> NMIBC |  |  | 2 |
| Total paired assessments |  |  | 107 |
| Discordance |  |  | 19/107 (17.8%) |
| MRI-to-definitive-pathology interval | 12 days (IQR 8-18) | 13 days (IQR 8-20) | 12 days (IQR 8-19); P = 0.514 |
| After excluding 14 inadequate TURBT cases: Clinical-radiologic AUC | 0.847 | 0.706 |  |
| After excluding 14 inadequate TURBT cases: VI-RADS AUC | 0.845 | 0.800 |  |
| After excluding 14 inadequate TURBT cases: Radiomics AUC | 0.909 | 0.836 |  |
| After excluding 14 inadequate TURBT cases: Combined AUC | 0.928 | 0.895 |  |

For paired assessments, cystectomy was the definitive reference standard. The 14-case sensitivity analysis excluded all patients whose TURBT specimen had inadequate detrusor-muscle representation.

Table S7. Validation-cohort operating characteristics of the combined nomogram at illustrative probability thresholds.

| **Threshold** | **Sensitivity** | **Specificity** | **PPV** | **NPV** |
| --- | --- | --- | --- | --- |
| 0.30 | 30/33 = 90.9% | 53/72 = 73.6% | 61.2% | 94.6% |
| 0.40 | 29/33 = 87.9% | 58/72 = 80.6% | 67.4% | 93.5% |
| 0.50 | 28/33 = 84.8% | 62/72 = 86.1% | 73.7% | 92.5% |

The thresholds are descriptive operating points and are not proposed as stand-alone treatment thresholds. PPV, positive predictive value; NPV, negative predictive value.

Table S8. Occupied grey-level bins after image normalization and fixed-bin-width discretization.

| **Sequence** | **Median number of bins** | **IQR** | **Range** |
| --- | --- | --- | --- |
| T2WI | 24 | 21-28 | 14-39 |
| DWI | 22 | 19-27 | 13-38 |
| ADC | 23 | 20-27 | 14-41 |

Normalization used I_norm = 100 × (I - μ)/σ with normalizeScale = 100, followed by fixed binWidth = 25.

Table S9. Documented MRI acquisition framework according to scanner.

Both 3.0-T systems followed the same institutional bladder mpMRI protocol framework throughout the study period. The table reports acquisition characteristics documented in the study materials and used to characterize scanner consistency.

| **Acquisition characteristic** | **Magnetom Skyra** | **Magnetom Verio** |
| --- | --- | --- |
| Field strength | 3.0 T | 3.0 T |
| Receive coil | 32-channel phased-array body coil | 32-channel phased-array body coil |
| T2-weighted imaging | High-resolution turbo-spin-echo; axial, sagittal and coronal planes | High-resolution turbo-spin-echo; axial, sagittal and coronal planes |
| Diffusion-weighted imaging | Axial DWI; at least three b-values, typically 0, 800 and 1,400 s/mm² | Axial DWI; at least three b-values, typically 0, 800 and 1,400 s/mm² |
| ADC maps | Automatically reconstructed from DWI | Automatically reconstructed from DWI |
| Dynamic contrast-enhanced imaging | Axial T1-weighted gradient-echo after standard-dose macrocyclic gadolinium | Axial T1-weighted gradient-echo after standard-dose macrocyclic gadolinium |
| Patient preparation | Moderate bladder filling according to departmental SOP | Moderate bladder filling according to departmental SOP |
| Protocol stability | Same institutional protocol framework maintained during 2019-2024 | Same institutional protocol framework maintained during 2019-2024 |

DWI, diffusion-weighted imaging; ADC, apparent diffusion coefficient; DCE, dynamic contrast-enhanced; SOP, standard operating procedure.

Figure S1. Patient flow and chronological cohort allocation.
